# Covariate linkage analysis of GAW14 simulated data incorporating subclinical phenotype, sex, population, parent-of-origin, and interaction

**DOI:** 10.1186/1471-2156-6-S1-S45

**Published:** 2005-12-30

**Authors:** Marian L Hamshere, Stuart MacGregor, Valentina Moskvina, Ivan N Nikolov, Peter A Holmans

**Affiliations:** 1 Biostatistics and Bioinformatics Unit, Cardiff University, Wales College of Medicine, Heath Park, Cardiff CF14 4XN, UK; 2 Department of Psychological Medicine, Cardiff University, Wales College of Medicine, Heath Park, Cardiff CF14 4XN, UK

## Abstract

**Background:**

We evaluate a method for the incorporation of covariates into linkage analysis using the Genetic Analysis Workshop 14 simulated data. Focusing on a randomly chosen replicate (42) we investigated the effect of the 12 subclinical phenotypes, sex, population, and parent-of-origin on the linkage signal from a model-free linkage analysis of Kofendrerd Personality Disorder.

**Results:**

We detected a linkage peak on chromosome 1, at about 175 cM, which varied depending upon individuals' status for subclinical phenotype b. A linkage peak on chromosome 3 (310 cM) was found not to depend upon subclinical phenotype status. Further peaks were found on chromosomes 5 (12 cM), 9 (4 cM), and 10 (95 cM), depending on the status of subclinical phenotypes a, k, and c/d/g, respectively.

**Conclusion:**

Retrospective comparison of our results with the simulation model showed correct identification of disease loci D1-5 on chromosomes 1, 3, 5, 9 and 10, respectively.

## Background

We chose to analyze all four populations of replicate 42 from the simulated data set. All analyses were performed without knowledge of the simulation model. The aim of the analysis was to utilize the information on the subclinical phenotypes of Kofendrerd Personality Disorder (KPD), sex, population, and parent-of-origin in a linkage analysis. Including covariates in the analysis allowed us to investigate models, such as locus heterogeneity, that give rise to different subclinical phenotypes within KPD. We present the results of our analyses and a retrospective comparison with the simulation model.

## Methods

We began by screening the genome for linkage to KPD. We performed separate scans of the microsatellites and the single-nucleotide polymorphism (SNP) data using the *Zlr *test statistic from ALLEGRO [1], with the "pairs" option and exponential model. Pedigrees with more than 17 individuals were trimmed to permit analysis with the software. We then examined the effect of the covariates on the linkage peaks. To do this we fitted subclinical phenotype, sex, population, and parent-of-origin status as covariates in a model-free linkage analysis of the microsatellite marker data. We also looked for interactions between linkage peaks using this approach.

### Linkage analysis using covariates

#### Likelihood construction

The multipoint likelihood of the marker data of an affected relative pair at any point in the genome is given by



where z_*j *_is the (unknown) probability that an affected relative pair share *j *alleles identically by descent (IBD), and *f*_*ij*_, are the prior and posterior (conditional on the observed marker data) probabilities that pair *i *shares *j *alleles IBD [2,3]. These were obtained for each pair at 1-cM intervals with and without parental specific allele sharing estimates using MERLIN [4] and ALLEGRO [1], respectively. Let *p*_*FS *_be the probability that a pair of affected full siblings share a given parental allele IBD. Following the suggestion of Rice [5,6], in the absence of a parent-of-origin effect the probabilities of sharing paternal and maternal alleles IBD were assumed to be equal and independent. Then z_0 _= (1 - *P*_*FS*_)^2^, z_1 _= 2 *p*_*FS *_(1 - *P *_*FS*_), and . Similar formulae apply for double-first-cousin pairs.

Other types of relative pair, *R*, can only share 0 or 1 allele IBD. For these, z_0 _= 1 - *P*_*R*_, z_1 _= *P*_*R*_, and z_2 _= 0 (where *P*_*R *_is the IBD probability for affected relative pairs of type *R*).

#### Inclusion of categorical covariates

The effect of a binary covariate on the IBD sharing probabilities may be investigated by modelling *P*_*R *_in a logistic regression framework including a 3-level factor β with levels corresponding to the status of the pair with respect to the covariate (-/-, -/+ or +/+, where - denotes absence and + presence of the covariate in an individual). That is,



where *O*_*R *_is a fixed offset, ensuring that *P*_*R *_takes the correct value for a relative pair of type *R *in the absence of linkage (i.e., all coefficients in the regression = 0). Under the null hypothesis of no covariate effect, α is a measure of the divergence of IBD from the null in the sample as a whole. The subscript *k *indexes the status of the particular relative pair with respect to the covariate. Multiple pairs from the same pedigree were analysed as if they were independent, with parameters α and β in common. To ensure identifiability of the parameters, β_-/- _was set to zero (making α a measure of IBD divergence from the null in -/- pairs). The degree of IBD sharing for the discordant (-/+) pairs was constrained to be less than or equal to the maximum IBD in the concordant pairs, to ensure that the model makes sense biologically. Each of the subclinical phenotypes (a - l) was modelled in this way, as was sex (male denoted by -, female denoted by +). Population membership was modelled as a four-level factor, with one level for each population (the first was set to zero). The total number of affected relative pairs in each category is shown in Table 1. One might expect a gene that modified the expression of a binary covariate (e.g., subclinical phenotype outcome) in individuals affected with KPD (but not KPD risk itself), to present increased sharing in -/- or +/+ pairs (or both), with -/+ pairs showing reduced sharing. A gene that acts to cause KPD with a particular set of covariate values (- or +) would cause increased sharing in either -/- or +/+ pairs, with the effects on IBD in the pairs of other types being unclear (dependent on penetrances, gene frequencies, etc.). Caution should be applied to the interpretation of the allele sharing estimates as the differences could arise from a number of reasons.

#### Inclusion of quantitative covariates

Locus × locus interactions between the peaks were investigated by including the estimated IBD sharing value for each pair at one location on a different chromosome (having subtracted the expected value in the absence of linkage) as a quantitative covariate in the logistic regression for IBD at the peak of interest [7]. This is then repeated for a number of locations in the region surrounding the locus being conditioned on to allow for the fact that linkage peaks are often some distance from disease loci [8]. The test statistic was taken to be the increase in maximum LOD score over the whole region investigated (covering both linkage peaks). For completeness, the hypothesis of an interaction between two peaks was investigated with two tests, i) peak 1 conditional on peak 2, and ii) peak 2 conditional on peak 1. In general, these give similar results.

#### Inclusion of parent-of-origin covariate

Finally, parent-of-origin effect was modelled in affected sibling pairs only by splitting the prior and posterior probabilities, *f*_*i1 *_and , of sharing 1 allele IBD into components reflecting whether the paternal or maternal allele was shared. The IBD probabilities for affected pairs were expressed in terms of IBD probabilities for the paternal (*p*_*pat*_) and maternal (*p*_*mat*_) alleles (e.g., *z*_2 _= *p*_*pat *_*p*_*mat*_), with the test statistic for parent-of-origin effect given by a likelihood-ratio test of *p*_*pat *_= *p*_*mat*_.

#### Test statistic and significance levels

To test for effects of categorical or quantitative covariates, the likelihood was maximized with respect to α alone at each position *x*, to give , and to both α and β, giving . The ratio of the maximum likelihoods on the chromosome, with and without the covariate of interest, gives a LOD score, which was used as the test statistic



We allowed the location of the maximum likelihood to change when the covariate was added. This reflects the fact that linkage peaks from standard analyses are often some distance from the true disease locus [8]. Incorporating the covariate may thus give a more accurate estimate of the disease locus location. Other test statistics are possible, for example the maximum point-wise likelihood ratio. However, the relative performance of these test statistics is unclear at present. Chromosome-wide significance levels were obtained by keeping the genotypes fixed and randomly permuting individual covariate values among the affected individuals. Pairwise covariate values were then calculated and the analysis repeated, thus significance levels reflect the dependency of pairs within a pedigree. To test for a parent-of-origin effect, the designations of paternal and maternal alleles were randomly swapped for all affected siblings in a sibship. If *n *replicates are generated in this manner, of which *r *give a test statistic greater than that in the actual data, the chromosome-wide *p*-value is estimated by (*r *+ 0.5)/(*n *+ 0.5).

For the test statistic chosen for this analysis, it was not possible to obtain a genome-wide significance level for covariate effects because this depends not only on the increase in LOD score given by the covariate, but also on the linkage evidence present without allowing for the covariate, i.e., based on . For example, an increase in LOD score of 2 to 3 is more significant than from 0 to 1 because the former is likely to occur by chance (in the absence of covariate effects) only in a linkage peak region, whereas the latter could occur anywhere on the chromosome. An estimate of genome-wide significance for a given chromosome, allowing for multiple testing, involves a joint Bonferroni-type adjustment for the relative length of the chromosome and the number of covariate tests conducted.

The subclinical phenotypes c, d, and g were indistinguishable in the affected individuals and e, f, and h were all present in the affected individuals and hence provided no useful information for analysis. Therefore, we have carried out 10 covariate analyses on each chromosome (subclinical phenotypes a, b, c, i, j, k, and l, sex, population and parent-of-origin). The interaction analyses were carried out between identified peak regions and hence were treated separately.

## Results

We found genome-wide significant linkage peaks on chromosomes 1 (max *Zlr *= 4.97 at 177 cM), 3 (max *Zlr *= 5.58 at 310 cM), 5 (max *Zlr *= 5.11 at 12 cM) and 9 (max *Zlr *= 6.04 at 4 cM). On chromosome 1 the peak was narrower with the 3-cM SNP map than with the microsatellite map, but this effect was not seen for the other peaks.

The linkage signal on chromosome 1 was found to increase substantially when the subclinical phenotype b was fitted as a covariate in the relative pair covariate linkage analysis, a LOD of 7.07 being increased to 14.29 (chromosome-wide *p *< 0.0001, genome-wide *p *= 0.0097). The linkage evidence appeared to come entirely from the +/+ pairs (IBD = 0.66, compared to 0.49, 0.48 from the -/-, -/+ pairs). A similar effect was found on chromosome 5 with the subclinical phenotype a (LOD increased from 4.90 to 10.05, chromosome-wide *p *< 0.0001, genome-wide *p *= 0.0096), with the linkage coming from the -/- pairs (IBD_-/- _= 0.62, IBD_-/+ _= 0.44, IBD_+/+ _= 0.51), and chromosome 10 (at 95 cM) with the subclinical phenotype c (LOD increased from 1.04 to 5.31, IBD_-/- _= 0.63, IBD_-/+ _= 0.43, IBD_+/+ _= 0.53, chromosome-wide *p *= 0.0004, genome-wide *p *= 0.063). On chromosome 9, the LOD increased from 7.65 to 18.13 with subclinical phenotype k (chromosome-wide *p *< 0.0001, genome-wide *p *= 0.0096), with increased sharing in both the -/- and +/+ pairs (IBD_-/- _= 0.63, IBD_-/+ _= 0.42, IBD_+/+ _= 0.67). No genome-wide significant effect of subclinical phenotype was observed on chromosome 3. No significant results were obtained for the analyses considering differences in IBD owing to sex, population, parent-of-origin, or interactions between the four identified linkage peaks. For each analysis, the maximum LOD score is presented in Table 2.

## Discussion

Retrospective comparison of our results with the simulation model showed correct identification of disease loci D1-5 on chromosomes 1, 3, 5, 9, and 10, respectively. D1 influences phenotypes P1 and P3, which both have subclinical phenotype b, confirmed by the increased sharing we observed in the b+/+ affected pairs. D2 influences all three phenotypes, P1-3, with one or two of the subclinical phenotype b and c in a somewhat complicated manner. D2 also influences subclinical phenotype k. We observed increased IBD in the k+/+ pairs (chromosome-wide *p *= 0.016), but this was not significant at the genome-wide level.

D2 and D3 together help to produce P2 and P3, with D3 also influencing subclinical phenotype a. We detected the association of subclinical phenotype a with D3, finding elevated sharing in the -/- pairs and decreased sharing in the -/+ pairs. D4 is related to P2 through subclinical phenotype c and P3 through b and c. D4 also influences subclinical phenotype k, which we observed through increased IBD sharing in pairs concordant for k.

No interactions were found between loci D1-4 when examining relative pairs concordantly affected for KPD in general, or even the relevant phenotype (P1-3). This is because the penetrances of the low-risk genotype combinations were set to zero, giving a multiplicative model for interactions. Under such models, IBD sharing at one locus is independent of that at the other [2]. The D1–D4 interaction could be detected by analyzing affected pairs to which exactly one member had P3 (a negative correlation in IBD was observed at the two loci). However, the D2–D3 interaction in P3 and the D1–D2 interaction in P1 were not detected by this method, due to the reduced penetrance of the relevant genotypes. Likewise, no linkage evidence was obtained at D6 (a modifying locus that affects the penetrance of phenotype P2), even when affected pairs discordant for P2 were analyzed. These results are consistent with the observation that affected relative-pair analysis has low power to detect locus-locus linkage interactions [7].

## Conclusion

From analyzing the data blind to the simulation model, there appear to be five susceptibility genes for KPD, located on chromosomes 1, 3, 5, 9, and 10. Those on chromosomes 5 and 10 appear to influence disease only in the absence of subclinical phenotypes a and c/d/g respectively. The locus on chromosome 1 influences disease only in individuals with subclinical phenotype b, whereas that on chromosome 9 appears to have two variants, one giving rise to the presence of subclinical phenotype k in affected individuals, the other to its absence. No subclinical phenotype was found to have a significant genome-wide effect on the linkage of KPD to chromosome 3, although k reached chromosome-wide significance. Even with knowledge of the simulation model, it was difficult to detect the locus-locus interactions, suggesting that affected relative pairs give little power for such analyses.

## Abbreviations

IBD: Identical by descent

KPD: Kofendrerd Personality Disorder

SNP: Single-nucleotide polymorphism

## Authors' contributions

All authors contributed to the statistical analysis and interpretation of the data, and to the drafting of this article.

## Figures and Tables

**Table 1:** The total number of affected relative pairs in each covariate category

	Covariate Categories					Covariate Categories		
			
Phenotype	-/-	-/+	+/+		Phenotype	-/-	-/+	+/+
A	517	161	74		i	527	211	14
B	129	180	443		j	561	173	18
c/d/g	165	186	401		k	268	221	263
e/f/h	0	0	752		l	602	122	28
	AI	DA	KA	NY				
Population	154	143	161	294	Sex	173	373	206

Subclinical phenotypes (and sex) are split into -/-, -/+ and +/+, where - and + indicate absent (male) and present (female), respectively. Population splits the data into four groups; AI (Aipotu), DA (Danacaa), KA (Karangar) and NY (New York City).

**Table 2:** Maximum LOD scores and number of parameters estimated for each covariate analysis on each chromosome

Covariate analysis^a^	Number of parameters	Maximum LOD score for each chromosome^b^									
		
		1	2	3	4	5	6	7	8	9	10
A	3	8.00	3.07	8.67	2.31	**10.05**	2.31	2.03	2.56	8.77	1.35
B	3	**14.29**	3.00	8.26	1.93	4.91	2.71	2.87	1.49	9.76	1.67
c/d/g	3	8.11	2.67	8.45	1.82	6.50	2.44	1.71	2.60	8.75	**5.31**
e/f/h	3	7.07	2.03	7.49	0.52	4.90	1.53	0.71	0.59	7.65	1.04
I	3	7.72	2.20	8.41	1.64	7.34	2.62	1.33	2.07	8.45	1.51
J	3	7.22	2.94	7.73	2.83	6.97	3.37	1.28	1.74	9.30	1.57
K	3	7.35	2.69	9.43	2.38	5.46	2.24	2.11	2.77	**18.13**	1.33
L	3	7.53	2.61	9.38	2.01	5.25	2.01	1.28	1.58	9.92	1.65
Sex	3	7.30	4.18	7.67	2.26	5.40	1.81	1.26	1.74	8.01	2.09
Population	4	8.55	3.89	7.73	1.62	5.72	2.67	2.49	1.88	9.25	3.42
Univariate (ARP)	1	7.07	2.03	7.49	0.52	4.90	1.53	0.71	0.59	7.65	1.04
Parent-of-origin (ASP)	2	5.77	2.42	8.05	0.57	3.90	2.31	1.88	1.21	7.48	2.00
Univariate (ASP)	1	4.84	1.75	7.14	0.41	3.75	2.29	0.62	0.45	7.35	1.67

^a ^All analyses, except for parent-of-origin, were based on affected relative pairs (ARP). Parent-of-origin was an affected sibling pair (ASP) analysis.

^b ^The LOD scores marked in **bold **indicate the analyses that showed an increase in maximum LOD score from the baseline univariate to the covariate LOD that reached genome-wide significance. Chromosome 10, subclinical phenotype c/d/g was genome-wide significant at *p *= 0.063 and has also been indicated in **bold.**
